# Evaluation of Bufadienolides as the Main Antitumor Components in Cinobufacin Injection for Liver and Gastric Cancer Therapy

**DOI:** 10.1371/journal.pone.0169141

**Published:** 2017-01-12

**Authors:** Xiaolu Wei, Nan Si, Yuefei Zhang, Haiyu Zhao, Jian Yang, Hongjie Wang, Lianmei Wang, Linyu Han, Baolin Bian

**Affiliations:** 1 Institute of Chinese Materia Medica China Academy of Chinese Medical Sciences, Beijing, China; 2 China Resources Sanjiu Pharmaceutical Co., Ltd, Shenzhen, Guangdong Province, China; State University of New York, UNITED STATES

## Abstract

**Background:**

Cinobufacin injection, also known as huachansu, is a preparation form of Cinobufacini made from Cinobufacin extract liquid. Despite that Cinobufacin injection is shown to shrink liver and gastric tumors, improving patient survival and life quality, the effective components in Cinobufacin remain elusive. In this study, we aim to screen antitumor components from Cinobufacin injection to elucidate the most effective antitumor components for treatment of liver and gastric cancers.

**Materials and Methods:**

High performance liquid chromatography (HPLC) and LC-MS/MS analysis were used to separate and determine the components in Cinobufacin injection. Inhibition rates of various components in Cinobufacin injection on liver and gastric cancer cells were determined with MTT assay; Hepatocellular carcinoma and gastric cancer models were used to assess the antitumor effect of the compounds *in vivo*.

**Results:**

The major constituents in Cinobufacin injection include peptides, nucleic acids, tryptamines and bufotalins. MTT assay revealed that bufadienolides had the best antitumor activity, with peptides being the second most effective components. Bufadienolides showed significant inhibition rates on gastric and hepatocellular tumour growth *in vivo*.

**Conclusion:**

Bufadienolides are the most effective components in Cinobufacini injection for the treatment of liver and gastric cancers. This discovery can greatly facilitate further research in improving the therapeutic effects of Cinobufacin injection, meanwhile reducing its adverse reaction.

## Introduction

Cinobufacin is a water-soluble extract from the dried skins of the toad Bufo bufo gargarizans Cantor, Bufo melanostictus Schneider, and Bufo raddei Sauch. Cinobufacin has been used in China as an effective traditional Chinese medicine to treat conditions like swelling, pain, and heart failure for thousands of years [1]. Cinobufacin injection, a preparation form of Cinobufacin made from Cinobufacin extract liquid, has been approved by the Chinese State Food and Drug Administration (SFDA) (ISO9002) and widely used to treat a variety of cancers in China. Clinical evidences indicated that Cinobufacin injection could be used in combination with chemotherapy or radiotherapy to enhance the antitumor efficacy [2, 3]. The antitumor effects of Cinobufacin injection originate from its ability to inhibit cancer cell proliferation and differentiation, induce apoptosis, and enhance immune responses against cancer [2, 3]. Its therapeutic effect is especially pronounced in liver cancer, with the tumor inhibition efficiency up to 44.4% [4]. More importantly, no dose-limiting toxicities, including cardiovascular, hematologic, gastrointestinal and mucocutaneous toxicities could be observed for Cinobufacin injection, even with the dosage up to eight times higher than what is typically used in China.

Alkaloids, including 5-serotonin, bufotenine, bufothionine, and dehydrogenation bufothionine nitrogenous substances, which comprise >1/10 of Cinobufacin, were once considered the main antitumor components [5]. At present, the quality control of Cinobufacin is mainly based on the total alkaloids content [6]. However, no clear evidences exist on the effectiveness of alkaloids in cancer suppression. In addition, the indole components in alkaloids, such as including 5-HT, bufotenine, toad poison and thiophene warns, induce strong stimulation of vascular wall, causing pain and vasoconstriction [7–9]. Thus, it is necessary to re-investigate the constituents of Cinobufacin injection, clarifying the material basis of its antitumor effects, to further increase the therapeutic efficacy of Cinobufacin injection and decrease its adverse effects.

Besides alkaloids, other major constituents of Cinobufacin include nucleoside [10], peptides [11] and bufadienolides [12]. Peptides in Cinobufacin injection, with molecular weight ranging from 0.7 to 1.5 kDa, have shown certain antitumor effect [13]. Bufadienolides exist at lower levels (5mg/L) in Cinobufacin injection, and include bufalin, cinobufagin, resibufogenin, bufotalinic, bufagin, telocinobufagin and other toadpoison steroidal diene compounds. It is suggested that bufadienolides in Cinobufacin may be responsible for the antitumor effects of Cinobufacin injection, but no direct evidences have been reported [14]. Therefore, we applied chromatography technologies to systematically screen components in Cinobufacin injection. The four components in Cinobufacin injection, including alkaloids, nucleosides, peptides and bufadienolides, were enriched and tested for their antitumor properties *in vitro*. As expected, bufadienolides exhibited the best antitumor effects *in vitro*, which is further verified by their prominent effects in suppressing hepatocellular and gastric tumor growth. Considering the huge unmet clinical need of an effective therapy against liver and gastric cancers, the use of Cinobufacin injection, in combination with western medicines, such as Docetaxel, holds promise to effectively improve patient survival and life quality [15, 16]. By identifying the main antitumor agent in Cinobufacin injection, this work could potentially benefit efforts in enhancing the therapeutic effects of Cinobufacin injection, while reducing its adverse effects.

## Materials and Methods

### Materials

Cinobufacin extract liquid (Lot: 131101/01) and Cinobufacin injection (500 mg/ml) (Lot: 131101–1) were kindly provided by Anhui JingChan Pharmaceutical Co., Ltd (Anhui, China). 5-fluorouracil and cyclophosphamide were purchased from Sigma Biological Engineering Technology (Saint Louis, Missouri, USA).

Analytical grade methanol and methylene chloride (Merck, Darmstadt, Germany) were used for preparing samples and standard solutions. HPLC grade acetonitrile (Merck, Darmstadt, Germany), C18 chromatographic column (Thermo Scientific, Tewksbury, MA, USA), HW-40C gel (Tosoh Corporation, Tokyo, Japan). Deionized water generated from a Milli-Q water system (Millipore, Bedford, MA, USA) were used for the preparation of mobile phase. Dimethyl sulfoxide (DMSO) and 3-(4,5-dimethylthiazol-2-yl)-2,5- diphenyltetrazolium bromide (MTT) were purchased from Sigma Aldrich (Saint Louis, Missouri, USA). RPMI-1640 medium, DMEM/F12 medium, fetal bovine serum, penicillin, streptomycin, and PBS were purchased from Gibco (Grand Island, NY, USA).

The chemicals used in the buffers and other solutions were all of analytical grade. All drugs and reagents were prepared immediately before use.

### Cells and animals

Cell lines used in this study include: human hepatocellular carcinoma cell lines BEL-7402 and HepG-2, murine hepatoma cell line H-22, human gastric cancer cell lines BGC-823 and MKN-45, and murine gastric cell line MFC. All cell lines were obtained from the Cell Resource Center, IBMS, CAMS/PUMC (Beijing, China).

K.M mice, ICR mice and BALB/c-nu nude mice were purchased from Vital River Experimental Animal Technology Co., Ltd. (Beijing, China License key: SCXK(Jing)2012-0001). The animals were housed in an environmentally controlled facility maintained at room temperature with relative humidity of 40–70%. The facility was maintained with a 12-hour light/dark cycle, and free access to food and water. The animals and experiments were conducted under the specific pathogen-free conditions. The animals were allowed to acclimatize themselves to the colony for 3 days before the experiments began and were randomly assigned to different groups. The animal management procedures and the experimental protocol were approved by the Institutional Animal Care and Use Committee (IACUC) of Institute of Chinese Materia Medica in China Academy of Chinese Medical Sciences. At the same time, all animal care and use procedures were performed according to Principles of Laboratory Animal Care.

### The isolation and enrichment of the effective fractions in Cinobufacin injection

The effective fractions in Cinobufacin injection were prepared as reported previously[17]. In short, the aqueous extract of toad skin was first precipitated by alcohol. Then the supernatant was purified and enriched by HW40-C gel column chromatography, resulting in separation of bufadienolides, alkaloids, nucleoside and peptides.

### The identification of effective fractions

The identification of fractions from Cinobufacin was performed with an ultimate 3000 LC system coupled with an LTQ Orbitrap mass spectrometer (Thermo-Velos Pro double pressure linear ion trap tandem Orbitrap mass spectrometry, Bermen, Germany) via an ESI interface as described previously[13]. The chromatography system consisted of an autosampler, a diode-array detector, a column compartment. Xcalibur, Metworks and Mass Frontier 7.0 software packages were used for data collection and data analysis.

The ESI source parameters were used as follows: capillary temperature, 380°C; source voltage and ispray voltage, 5 kV; sheath gas (N2) flow, 35 psi; and aux gas flow, 10 psi. The data was monitored in the positive ionization mode with a mass resolution of 30,000. The LC-MS/MS experiments were set as data-dependent scans.

Liquid chromatographic separations of the analysts were performed using a Thermo Diamonsil@C18 column (4.6 mm× 250 mm, 5 μm). The mobile phase consisted of 0.1% formic acid in water (solvent A) and acetonitrile (solvent B). The gradient elution was as follows: 0–10 min, 5% to 25% B; 10–45 min, 25–46%B; 45–60 min, 46%-60% B; 60–65 min, 60%-100% B; 65–75 min, 100% B for equilibration of the column. The flow rate was 0.5 mL/min. The injection volume was 5 μL. The wavelength was set at 296 nm. The column oven was set at 30°C.

### Cell culture

Human hepatocellular carcinoma cell line BEL-7402 and human gastric cancer cell lines BGC-823 were cultured in DMEM/F12 medium containing 10% heat-inactivated fetal bovine serum (FBS) and 1% penicillin and streptomycin in a humidified atmosphere with 5% CO2 at 37°C using a water jacket type CO2 cell culture box (Sanyo, Japan). Cells were routinely passaged every 48–72 hours and cell samples used were all in the logarithmic growth phase.

### MTT assay

The cell proliferation was evaluated using a MTT colorimetric assay. Cells were cultured till 80% confluency in flask and dissociated from bottom by 0.25% trypsin containing 2% EDTA. Before performing MTT assay, BEL- 7402 (5000 cells/well) and BGC-823 cell lines (5500 cells/well) were seeded into 96-well plates (Costar, 3596) with culture medium, and incubated for 24 h. Then cells were treated with 5-FU and various concentrations of four effective fractions in Cinobufacin injection (bufadienolides, alkaloids, nucleoside and peptides) for 48h. At the end of the treatment, 20 μL of MTT reagent solution (5 mg/ml in PBS) were added to each well followed by incubation for an additional 4 h at 37℃. After the medium and MTT were removed, 200 μL DMSO was added to each well and placed on a plated shaker for 5min at room temperature in order to dissolve water-insoluble formazan. Then the spectrophotometric absorbance of the samples at 490nm wavelength was measured by Varioskan Flash microplate reader (Thermo Scientific, Tewksbury, MA, USA). IC_50_ values (concentration of drug that inhibits cell growth by 50%) are calculated for each component in Cinobufacin using SPSS17.0. In addition, the IC_50_ values of these compounds on L-02 normal liver cell lines were acquired using the same method. The inhibition rate (IR) was calculated as the following formula: percentage of inhibition = [1-(mean OD of experimental sample/mean OD of the control group)]×100%.

### Acute toxicity study

In order to determine the LD50 (amount of bufadienolides that kill 50 percent of testing mice) of bufadienolides, K.M mice (20±2g) were assigned randomly to one control and six treatment groups, with each group containing 5 male and 5 female mice. The treatment group were intraperitoneally injected with different concentrations of bufadienolides (10, 14, 16, 20, 24, 28 mg/kg in saline). The control group received saline (10 ml/kg) by gavage. During treatment, the number of deaths, mouse body weight and behaviors were recorded daily for 14 days.

### Construction of tumor models and treatment procedures

For murine tumor models, H22 mice hepatoma cells and MFC mice gastric carcinoma cells were used to initiate tumors. H22 and MFC Cells (ascites tumor/suspension) were diluted 1:3 with saline. 0.2ml cell suspension was intraperitoneally injected into ICR mice. After 7–10 days, tumor bearing mice were sacrificed to collect little ascites. Then, the ascites with >75% viable cells were diluted 1:4 with pre-cold saline as tumor suspension. Each mouse was inoculated with 0.2 mL tumor suspension in the right armpits. 24 h after inoculation, mice were randomly grouped, with each group containing 5 male mice and 5 female mice. As a positive control, one group of mice were intraperitoneally injected with Cyclophosphamide (CTX) once. Three groups of mice were intraperitoneally injected with different doses (3 mg/kg, 1.5 mg/kg and 0.75 mg/kg) of bufadienolides once per day for 7 consecutive days while the blank group was injected with saline for the same duration. 24 h after the last injection, mice were weighed before being sacrificed. Tumor weight was also measured to evaluate tumor inhibition efficacy of each compound.

To construct human hepatocellular carcinoma and human gastric tumor xenografts. The human HepG-2 hepatocellular carcinoma cells and human MKN-45 gastric cancer cells were cultured till logarithmic growth phase. Trypan blue stain assay was used to examine the cell viability. A cell suspension (5×10^6^ cells/mL) with up to 95% viable cells was prepared, and 0.2ml cell suspension was subcutaneously inoculated in each BALB/C-nu nude mice on the right side of axillary. The tumors were serially passaged 2–3 times in vivo to ensure best tumorigenicity. During the last implantation, mice were randomly grouped when the tumor size reached 300 mm^3^, with each group containing 3 male and 3 female mice. As a positive control, one group of mice received Cinobufacin injection, and two groups of mice received intraperitoneal injection of bufadienolides at different doses (2 mg/kg and 0.5 mg/kg) three times a week for 4 weeks. Blank group received saline for the same duration. During treatment, 0.02mm precision vernier caliper was used to measure the long diameter (a, mm) and short diameter (b, mm) of tumor to calculate tumor volume (V, mm^3^), relative tumor volume (RTV) and relative tumor growth rate (T/C%) according to the following equations:

$$\begin{array}{l} V=a\times b^{2}\times 0.5;\\ RTV=V_{t}/V_{0}\;(V_{0}:\;the\;volume\;before\;injection;\;V_{t}:\;the\;volume\;during\;treatment);\\ T/C\%=TRTV/CRTV\times 100\%\;(TRTV:Treatment\;group\;RTV;\;CRTV:\;Blank\;group\;RTV); \end{array}$$

Mouse weight was also measured twice a week to calculate tumor growth inhibition rate according to the following equation: Tumor growth inhibition rate = (1–T/C) ×100%, where T is average tumor weight of treatment groups and C is average tumor weight of the blank group.

### Statistical analysis

Statistical analysis was performed using the SPSS 16.0 software. All experiments were performed in a minimum of triplicate and the results were expressed as means±standard deviation (SD). Differences between two groups were evaluated by Student's t-test. Comparisons among multiple-group were evaluated by one-way Analysis of Variance (One-Way ANOVA) and LSD t-test was used for multiple comparisons. Differences with P<0.05 (*), P<0.01 (**), and P<0.001 (***) were considered statistically significant.

## Results

### Characterization of different effective fractions in Cinobufacin injection

**Fig 1:**
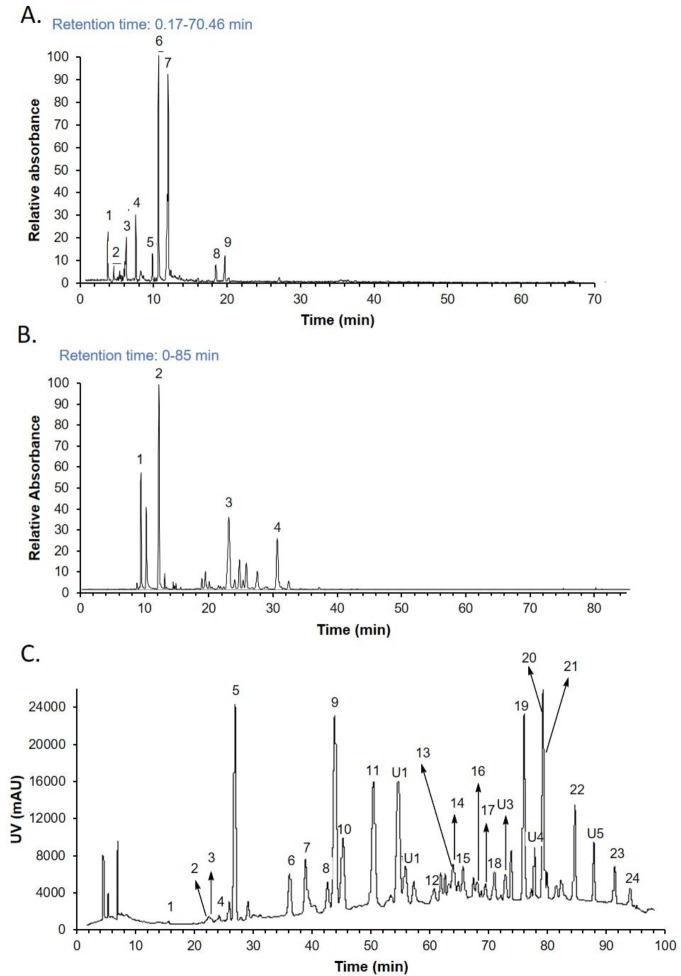
Chromatographic characterization of Cinobufatin components. A. total ionic chromatogram of nucleic acids. B. total ionic chromatogram of tryptamines. C. Ultraviolet diagram of bufotalins. Identification of peaks are shown in supplementary tables.

Peptides, nucleic acids, tryptamines, and bufotalins, were obtained and identified as four major fractions in Cinobufacin injection after gel chromatography separation. Peptide fraction was identified using biuret test. Mass spectrum analysis indicated that the molecular weight of the peptides was 2000–5000 Da. Nucleic acids and tryptamines fractions were identified using total ionic chromatogram, as shown in **Fig 1A** and **Fig 1B** respectively. Bufotalins were identified by ultraviolet diagram, as shown in **Fig 1C**. Identifications of individual peaks in **Fig 1A**, **Fig 1B**, and **Fig 1C** are shown in **S1, S2 and S3 Tables** respectively. This result is in consistency with previously published studies [18–20].

### Bufadienolides was identified as the most effective antitumor components among the four constituents from Cinobufacin

**Fig 2:**
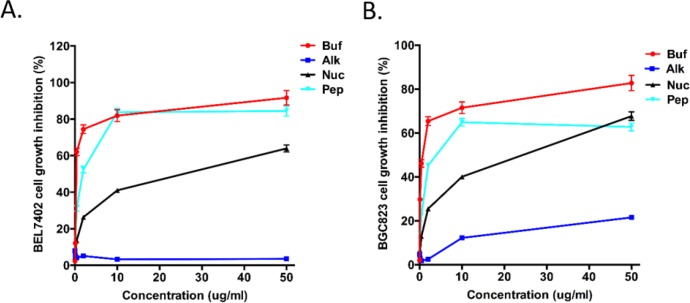
Evaluation of antitumor effects of different Cinobufacin components. Growth inhibition effects of different concentrations of bufadienolides (Buf), alkaloids (Alk), nucleosides (Nuc), and peptides (Pep) extracted from Cinobufacin on BEL-7402 (A) and BGC-823 cells (B). Cells was monitored for 48hrs after adding different Cinobufacin extracts.

To screen for components with best antitumor activity, bufadienolides, alkaloids, nucleoside and peptides in Cinobufacin injection were tested for their inhibition rate on human hepatocellular carcinoma cell BEL-7402 and human gastric cancer cell BGC-823. Cells were exposed to the extracts of the four components using 5-Fu as a positive control. After 48h exposure, MTT assay was used to verify the effect of the four components on cancer cells proliferation. The assays revealed that bufadienolides had the most effective antitumor activity than the other three components, with peptides being the second most effective components. Alkaloids has almost no anti-cancer activity. Bufadienolides displayed a dose-dependent inhibition on BEL-7402 and BGC-823 cells with an IC_50_ of 0.28±0.05 and 0.49±0.08 μg/ml, respectively (see **Fig 2** and **Table 1**), which are much lower than other constituents of Cinobufacin. Based on these results, we concluded that bufadienolides are the most effective component in Cinobufacin and proceeded to test their antitumor effect in vivo.

**Table 1:** IC_50_ of Cinobufacin components.

Name	IC_50_ (μg/ml)	
	BEL7402	BGC823
Bufadienolides	0.28±0.05	0.49±0.08
Alkaloids	>50	>50
Nucleoside	13.92±5.67	25.38±4.19
Peptides	1.88±0.55	4.96±1.28
5-Fu	0.20±0.02	0.42±0.02

### Acute toxicity study

Bufadienolides extracted from Cinobufacin were intraperitoneally injected in mice at different doses ranging from 10mg/kg to 28 mg/kg, followed by monitoring mice reaction for 14 days. At intermediate doses (14 mg/kg to 20 mg/kg), the mice exhibited an increased breathing rate, limb rigidity, opisthothonos, shaking, intense struggle, and the body reaction disappeared after drug withdraw. With high doses (>20mg/kg), mice died in 10–15 min. The dose of 10 mg/kg did not produce any signs of acute toxicity in the treated animals and the drug tolerance in female mice was slightly stronger than male (**Table 2**).

**Table 2:** The Acute toxicity study of bufadienolides on KM mice (n = 10).

Dosage	Number	Survival number		
		♂	♀	Total
10mg/kg	10	5	5	10
14mg/kg	10	4	5	9
16mg/kg	10	4	4	8
20mg/kg	10	2	3	5
24mg/kg	10	1	2	3
28mg/kg	10	0	0	0
Control	10	5	5	10

### The effect of bufadienolides on the inhibition of murine liver and gastric cancer

**Fig 3:**
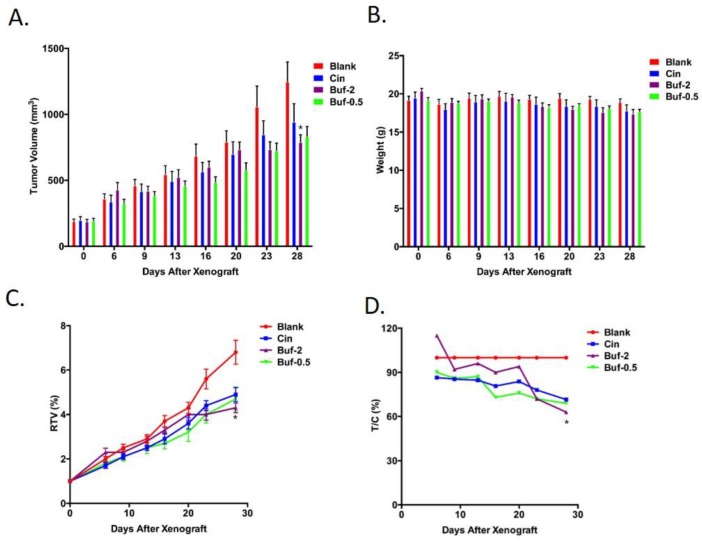
Cinobufacin (3.4mg/kg) and bufadienolides (2 mg/kg and 0.5 mg/kg) were evaluated on their influence on tumor volume (A) and nude mice weight (B) of MKN-45 gastric tumor model. The drug's influence on relative tumor volume (RTV, shown in C) and relative tumor growth rate (T/C (%), shown in D) of MKN-45 are were also presented. *: P<0.05 compared to Cinobufacin group.

As shown in **Fig 3** and **Table 3,** bufadienolides had significant inhibition of H22 and MFC murine tumor growth at the dose of in 3 mg/kg and 1.5 mg/kg. The tumor inhibition rate with 3 mg/kg bufadienolides was 32.2% and 33.8% for H22 and MFC tumor respectively, and the tumor inhibition rate with 2mg/kg bufadienolides was 29.5% and 25.6% respectively. In comparison, 3.4mg/kg Cinobufacin only resulted in 17.1% and 14.3% inhibition on H22 and MFC tumor respectively.

**Table 3:** Effect of bufadienolides on H22 and MFC tumors.

Dosage	Group	H22		MFC	
		Tumor weight (g)	Inhibition (%)	Tumor weight (g)	Inhibition(%)
Blank		1.737±0.378	—	3.035±0.646	—
Bufadienolides	3 mg/kg	1.177±0.579*	32.2	2.011±0.440**	33.8
Bufadienolides	1.5 mg/kg	1.224±0.387**	29.5	2.259±0.391*	25.6
Bufadienolides	0.75 mg/kg	1.506±0.476	13.3	2.714±0.361	10.6
Cinobufacin	3.4 ml/kg	1.440±0.435	17.1	2.602±0.742	14.3
CTX	30 mg/kg	0.253±0.171***	85.4	1.077±0.261***	64.5

***P<0.001.

** P<0.01.

*P<0.05 compared to Cinobufacin group.

### The effect of bufadienolides on the inhibition of human liver and gastric tumor xenografts

As shown in **Fig 3**, both Cinobufacin injection and bufadienolides significantly inhibited HepG-2 tumor as reflected in a slower increase of tumor volume. Notably, HepG-2 tumor volume on mice treated with 2mg/kg bufadienolides was less than half of tumor volume on nontreated mice (blank) at the end of six weeks. This agrees with the finding that 2mg/kg bufadienolides significantly decreased tumor weight compared to control group, leading to an inhibition rate of 57.6% (**Table 4**). This trend can also be reflected in RTV and T/C% (**Fig 3**) that 2mg/kg bufadienolides showed the best antitumor effect. Bufadienolides of 0.5 mg/kg also showed significantly higher inhibition effect than Cinobufacin (P<0.001), especially in the early phase of tumor growth (**Fig 3D**). Despite the inhibitive effect of Cinobufacin injection and bufadienolides on HepG-2 tumor, both of them did show significant effect on mice weight. 0.5mg/kg bufadienolides had almost no effect on mouse weight.

**Table 4:** Effect of bufadienolides on HepG-2 and MKN-45 tumors.

Dosage	Group	HepG-2		MKN-45	
		Tumor weight(g)	Inhibition(%)	Tumor weight(g)	Inhibition(%)
Blank		1.565±0.219	—	1.038±0.295	—
Cinobufacin	3.4 ml/kg	1.143±0.403	27.0	0.800±0.203	23.0
Bufadienolides	2.0 mg/kg	0.659±0.206***	57.9	0.696±0.059*	33.0
Bufadienolides	0.5 mg/kg	1.109±0.373*	29.1	0.870±0.142	16.2

***P<0.001.

** P<0.01.

*P<0.05 compared to Cinobufacin group.

**Fig 4:**
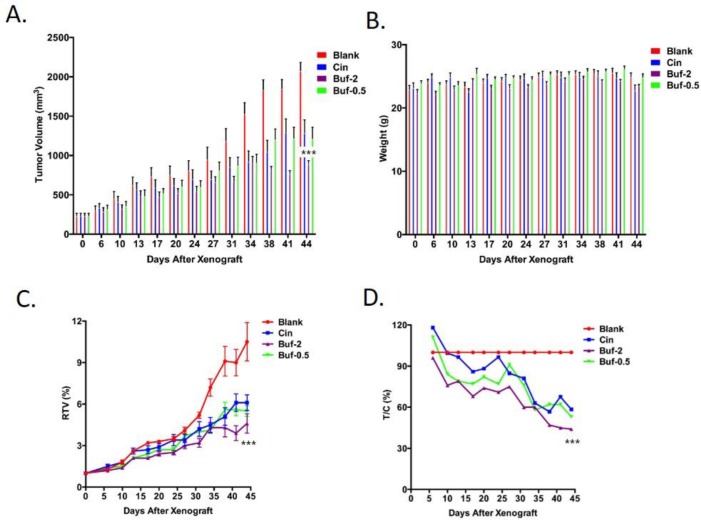
Cinobufacin (3.4mg/kg) and bufadienolides (2 mg/kg and 0.5 mg/kg) were evaluated on their influence on tumor volume (A) and nude mice weight (B) of HepG-2 liver tumor model. The drug's influence on relative tumor volume (RTV, shown in C) and relative tumor growth rate (T/C (%), shown in D) of HepG-2 are were also presented. ***: P<0.001 compared to Cinobufacin group.

Similar trend was also seen on MNK45 tumor. Bufadienolides of 2mg/kg exerted significantly higher antitumor effect than Cinobufacin group at the end of the 4-week treatment (P<0.05), with a tumor inhibition rate of 33% (**Fig 4A–4D**). No significant effect can be observed on mouse weight.

## Discussion and Conclusion

Liver and gastric cancer are the most common malignancies worldwide with a high incidence and mortality rate. While surgery is currently the first choice for initial treatment of these cancers, many patients are diagnosed with cancer with advanced stages, making them unsuitable for surgery. For example, early clinical symptoms of gastric cancer are easily overlooked, so early diagnosis is difficult. According to the International Union against cancer (UICC) and the United States Joint Committee (AJCC), about 65% gastric cancer patients belong to the terminal stage (T3/T4) in the United States, and 85% patients possess lymphatic metastasis. In China, the number of clinical gastric cancer patients at terminal stage is more than 70%. Therefore, treatment of these patient is only based on radiotherapy or chemotherapy.

One drawback of current drugs for chemotherapy is that they are toxic not only to tumors but also to normal tissue. Thus, patients’ life quality is severely damaged despite the limited effect of chemotherapy in prolonging survival. Traditional Chinese medicine (TCM) is a treasured natural repository, in which effective therapeutic drugs could be potentially discovered. Cinobufacin injection has been demonstrated as an effective drug in various cancers. More importantly, very little toxic effects can be observed with Cinobufacin injection. However, further development of Cinobufacin is hindered by the fact that the active ingredients in Cinobufacin is unclear. Quality control of Cinofacin is therefore difficult. Recently, a randomized phase II study demonstrated that Cinobufacin injection, when combined with gemcitabine, failed to improve the outcome of patients with locally advanced and/or metastatic pancreatic cancer [21]. This is partly due to the insufficient efficacy of Cinobufacin injection in current formulation. Development of more potent drug formulation demands a clear knowledge of the active components in Cinobufacin, and identification of their putative biomarkers.

In this study, we demonstrated that bufadienolides in Cinobufacin are the most effective antitumor components. MTT assay revealed that the IC_50_ value of bufadienolides was much lower than other components, suggesting that bufadienolides could contribute to tumor suppression despite its low concentration in Cinobufacin. Also, consistent with previous results, peptides showed certain antitumor efficacy. Surprisingly, alkaloids had no effect on tumor cells. Enriched bufadienolides, at doses lower than Cinobufacin (3mg/kg, 2 mg/kg and 1.5 mg/kg), demonstrated significantly higher tumor inhibition than Cinobufacin injection (3.4 mg/kg). This inhibition effect was reflected by a slower rate of tumor growth in mice injected with bufadienolides. Meanwhile, body weight was not affected, demonstrating that bufadienolides exerted no toxic effects on mice. In addition, the fact that 0.75mg/kg bufadienolides (higher than bufadienolides content in 3.4 mg/kg Cinobufacin), was not able to provide higher therapeutic efficacy than 3.4 mg/kg Cinobufacin implied that other components in Cinobufacin injection, for example peptides, may synergistically contribute to Cinobufacin’s effects. Considering that the highest dose we used was 3 mg/kg, dose escalation is possible to achieve even higher tumor inhibition effect since acute toxicity study indicated that 10 mg/kg dosage wouldn’t cause any adverse effect on mice.

In sum, bufadienolides are the main material basis in Cinobufacin injection to treat gastric and liver cancer. The use of bufadienolides, instead of alkaloids, would enhance the antitumor effect of Cinobufacin, as well as minimize adverse effects associated with alkaloids. This narrows down the possible active compounds within Cinobufacin injection for selection of a single active chemical. Further research on screening bufadienolides components may give birth to one or more drugs for the treatment of liver and gastric cancer. This would also enable studies on elucidating mechanism of Cinobufacin injection in inhibiting tumor growth.

## Supporting Information

S1 Table S1 Table. (DOCX)

S2 Table S2 Table. (DOCX)

S3 Table S3 Table. (DOCX)

S1 File Graphical abstract. (PDF)

S2 File Highlights. (DOCX)
