# Supplementary material for: Evaluation of Bufadienolides as the Main Antitumor Components in Cinobufacin Injection for Liver and Gastric Cancer Therapy
Source: PLoS One. 2017 Jan 12;12(1):e0169141. doi: 10.1371/journal.pone.0169141 (PMC5231367; doi:10.1371/journal.pone.0169141)
Supplement: S1 Table — (DOCX) [file pone.0169141.s001.docx]

Table.S1

| NO. | Molecular weight | Molecular formula | Name |
| --- | --- | --- | --- |
| 1 | 152 | C5H5N5O | Guanine |
| 2 | 136 | C5H5N5 | Adenine |
| 3 | 113 | C4H4N2O2 | Uracil |
| 4 | 137 | C5H4N4O | Hypoxanthine |
| 5 | 153 | C5H4N4O2 | Xanthine |
| 6 | 243 | C9H12N2O6 | Uridine |
| 7 | 127 | C5H6N2O2 | Thymine |
| 8 | 268 | C10H13N5O4 | Adenosine |
| 9 | 284 | C10H13N5O5 | Guanosine |
