# Supplementary material for: Evaluation of Bufadienolides as the Main Antitumor Components in Cinobufacin Injection for Liver and Gastric Cancer Therapy
Source: PLoS One. 2017 Jan 12;12(1):e0169141. doi: 10.1371/journal.pone.0169141 (PMC5231367; doi:10.1371/journal.pone.0169141)
Supplement: S2 Table — (DOCX) [file pone.0169141.s002.docx]

Table.S2

| NO. | Molecular weight | Molecular formula | Name |
| --- | --- | --- | --- |
| 1 | 176 | C10H12ON2 | 5-hydroxytryptamine |
| 2 | 202 | C12H14ON2 | Demethylation bufotenidine |
| 3 | 218 | C13H18ON2 | Bufotenidine |
| 4 | 283 | C12H15O4N2 S | Bufothionione |
