# Supplementary material for: Evaluation of Bufadienolides as the Main Antitumor Components in Cinobufacin Injection for Liver and Gastric Cancer Therapy
Source: PLoS One. 2017 Jan 12;12(1):e0169141. doi: 10.1371/journal.pone.0169141 (PMC5231367; doi:10.1371/journal.pone.0169141)
Supplement: S3 Table — (DOCX) [file pone.0169141.s003.docx]

Table.S3

| No | Name | Holding time（min） | Molecular weight | Molecular formula |
| --- | --- | --- | --- | --- |
| 1 | Deacetylation of cinobufagin alcohol | 15.02 | 416 | C24H32O6 |
| 2 | 3- table- false toad venom extract | 21.94 | 416 | C24H32O6 |
| 3 | False toad venom extract | 22.23 | 416 | C24H32O6 |
| 4 | 16- to acetyl -19- oxo Hua bufotalin | 24.12 | 430 | C24H30O7 |
| 5 | Gamabufotalin | 26.05 | 402 | C24H34O5 |
| 6 | 3-table -Gamabufotalin | 35.88 | 402 | C24H34O5 |
| 7 | Hellebore alcohol | 38.51 | 418 | C24H34O6 |
| 8 | 3-table - Yuanhua toadpoison fine | 42.33 | 402 | C24H34O5 |
| 9 | Arenobufagin | 43.86 | 416 | C24H32O6 |
| 10 | Table 3- - arenobufagin | 44.99 | 416 | C24H32O6 |
| 11 | Hellebrigenin | 50.01 | 416 | C24H32O6 |
| U1 | Hellebrigenin isomers | 54.22 | 416 | C24H32O6 |
| U2 | 19- hydroxy bufalin | 55.30 | 402 | C24H34O5 |
| 12 | Cinobufagin alcohol | 60.75 | 458 | C26H34O7 |
| 13 | Toad it linen | 63.97 | 414 | C24H30O6 |
| 14 | 3- oxygen Daisha toadpoison fine | 64.05 | 414 | C24H30O6 |
| 15 | Deacetylation bufotalin | 65.52 | 402 | C24H34O5 |
| 16 | 19- oxygen generation Chinese bufotalin | 67.93 | 472 | C26H32O8 |
| 17 | The Argentina toad poison fine | 69.22 | 414 | C24H30O6 |
| 18 | 7β- hydroxy resibufogenin | 71.04 | 400 | C24H32O5 |
| U3 | Toad it Li Ning isomers | 72,86 | 414 | C24H30O6 |
| 19 | Yuanhua toadpoison fine | 76.05 | 402 | C24H34O5 |
| U4 | Dehydrogenation to acetyl cinobufagin | 77.82 | 396 | C24H28O5 |
| 20 | Deacetylation of cinobufagin | 79.06 | 400 | C24H32O5 |
| 21 | Bufotalin | 79.25 | 444 | C26H36O6 |
| 22 | Cinobufacini it spirit | 84.72 | 458 | C26H34O7 |
| U5 | Bufotalin isomers | 87.92 | 444 | C26H36O6 |
| 23 | Cinobufagin isomers | 91.46 | 442 | C26H34O6 |
| 24 | 3- keto - deacetylation of cinobufagin | 94.12 | 398 | C24H30O5 |
