# Supplementary figures and images for: Evaluation of Bufadienolides as the Main Antitumor Components in Cinobufacin Injection for Liver and Gastric Cancer Therapy
Source: PLoS One. 2017 Jan 12;12(1):e0169141. doi: 10.1371/journal.pone.0169141 (PMC5231367; doi:10.1371/journal.pone.0169141)

## Graphical abstract

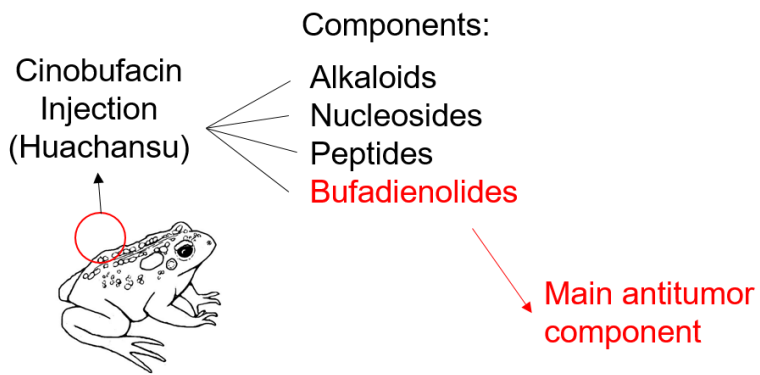

MKN-45 gastric cancer

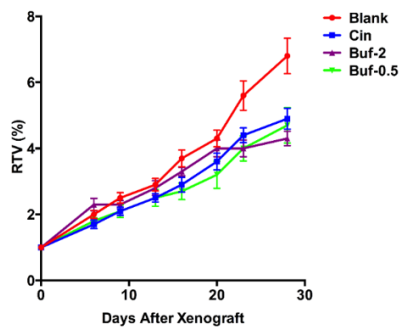

HepG-2 liver cancer

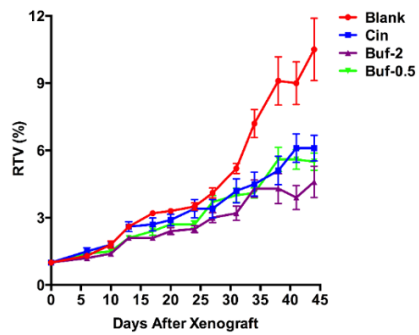

Supplement: S1 File — (PDF) [file pone.0169141.s004.pdf]
