# Supplementary material for: Evaluation of Bufadienolides as the Main Antitumor Components in Cinobufacin Injection for Liver and Gastric Cancer Therapy
Source: PLoS One. 2017 Jan 12;12(1):e0169141. doi: 10.1371/journal.pone.0169141 (PMC5231367; doi:10.1371/journal.pone.0169141)
Supplement: S2 File — (DOCX) [file pone.0169141.s005.docx]

**Highlights**

- We used chromatographic methods to separate and characterize main components in Cinobufacin injection;
- We evaluated the anti-tumour activity of these components in Cinobufacin injection in vitro and in vivo;
- We confirmed that bufadienolides are the main anti-tumor components in Cinobufacin injection;
- We evaluated the efficacy of bufadienolides in treating liver and gastric cancer in preclinical models;
